# Rise and fall: Results of a multidisciplinary study and 5-year long monitoring of conservation translocation of the European ground squirrel

**DOI:** 10.3897/BDJ.10.e83321

**Published:** 2022-06-17

**Authors:** Maria Kachamakova, Yordan Koshev, Dimitra-Lida Rammou, Svetoslav Spasov

**Affiliations:** 1 Institute of Biodiversity and Ecosystem Research at the Bulgarian Academy of Sciences, Sofia, Bulgaria Institute of Biodiversity and Ecosystem Research at the Bulgarian Academy of Sciences Sofia Bulgaria; 2 Aristotle University of Thessaloniki, Thessaloniki, Greece Aristotle University of Thessaloniki Thessaloniki Greece; 3 Bulgarian Society for the Protection of Birds, Sofia, Bulgaria Bulgarian Society for the Protection of Birds Sofia Bulgaria

**Keywords:** *Spermophiluscitellus*, *Microtushartingi*, endangered species, population reinforcement, morphometry

## Abstract

The current publication gives a detailed assessment of the results from a population reinforcement of a European ground squirrel's (*Spermophiluscitellus*) colony in south-eastern Bulgaria. The reinforcement was planned and implemented along with multidisciplinary research of the adaptation process (including radiotelemetry, parasitological study and assessment of the stress in the animals) and regular monitoring (yearly burrow counting). Although the donor and recipient populations were genetically similar, morphometrical data indicated variations in the body size probably due to the difference in population densities in the two sites. The monitoring revealed that the burrows were aggregated and there was a positive correlation in the spatial distribution of the ground squirrels’ burrow holes and the colonies of Harting's vole (*Microtushartingi*) - another social ground-digging rodent that co-inhabits the study area. The first results showed successful reinforcement according to the three classical evaluation criteria: the individuals survived the translocation process, they successfully reproduced and an initial population growth was observed, based on the burrow entrances’ count - from 36 in 2017 to 280 in 2020. In 2021, however, a considerable decline in the abundance of the population was recorded - 58.5% decline in the burrow number and 36% decline in the colony area. A decrease was also observed in the abundance of the Harting's voles' colonies. A review of all the collected information suggests it is unlikely that the decrease is due to helminth parasites, translocation stress or other behaviour issues. The most probable explanation is the bad weather conditions - unusually high rainfalls combined with relatively high temperatures in January 2021. In conclusion, we strongly emphasise the need for detailed and long-term monitoring after conservation translocation and careful evaluation of all the influencing factors before, during and after such actions.

## Introduction

Species conservation biology is a fast evolving and extremely challenging area of scientific research. In its essence, it is an interdisciplinary subject, based on natural and social sciences, merging these with natural resource management practices (Soulé 1985). One of its main tools is the translocation of individuals - the capture, transport and release of specimens from one location to another, aimed at improving the species’ conservation status through saving and reinforcing endangered populations, contributing to restoration of habitats, ecosystem functions (Swaisgood et al. 2019) and food chains (Koshev et al. 2019). The criteria for its success are diverse, but the most commonly used are: survival of the animals after release (phase I), settlement of the individuals at the site of release (phase II) and proven reproduction of the released animals (phase III) (Letty et al. 2003). However, further long-term assessment is crucial, as the situation can change significantly due to stochastic events (e.g. weather, parasites, predation) and some factors can have an impact at a later stage. Thus, an extended assessment scheme proposes two more phases - population growth (phase VI) and the ultimate goal - establishment of a viable population (IUCN 2013) that is large (hundreds of individuals) and has a good level of genetic diversity (phase V) (Source: Department of Conservation, NZ). However, that approach is related to the application of integrated monitoring during and following conservation translocation. The current study is an example of the implementation of such a procedure.

The European ground squirrel (known also as European souslik, *Spermophiluscitellus*) is a medium-sized rodent living in colonies in the open uncultivated lands of South-eastern and Central Europe. The species population number and range has declined by up to 50% according to IUCN (Hegyeli 2020), mainly due to intensification and use of chemicals in agriculture, reduction of pasturing livestock and shrubs and forest encroachment after grassland abandonment (Valkó et al. 2018). In consequence in 2020, the IUCN classified the European ground squirrel as an endangered species (Hegyeli 2020). Efforts for its conservation have been made along its range and a significant experience in their re-introduction has been gained in Central Europe over the last 35 years (Hapl et al. 2006, Balaz et al. 2008, Matějů et al. 2010, Gedeon et al. 2011, Matějů et al. 2012, Tokaj et al. 2012, Löbbová and Hapl 2014). Most of these actions are implemented in the northern part of the species’ range – Czech Republic, Slovakia, Hungary and Poland. Although the translocations are relatively common, they are not always successful – the reproduction stage was reached in only 50% of the cases (Matějů et al. 2012). The lessons learnt are rarely efficiently shared with the conservation and scientific community. The data on the translocations are often published only in local conference proceedings or were not published at all (Matějů et al. 2010). This has particularly bad implications for management, as these actions are, in general, costly and complex. Even if the data on the translocation itself become public, often the long-term monitoring is missing and it is not possible to assess the final result (Matějů et al. 2010, Koshev et al. 2019). As for the southern part of the range, several re-introductions and reinforcement were applied only in the last decade in Bulgaria (Koshev et al. 2019). The European ground squirrel is assessed as vulnerable by the Red Data Book of Bulgaria (Stefanov 2015), but only its habitats are protected under the Bulgarian legislation in the framework of the Natura 2000 network of protected sites. The data show a decrease in the range and abundance of the species since 2008. Pilot studies in the Natura 2000 site “Zapadna Strandzha” (BG0002066) in south-eastern Bulgaria confirmed an unfavourable trend on a local level - the last local colony has a critically low number of individuals despite the optimal conditions of the habitat. In order to prevent this extinction, urgent conservation actions were taken in the area. These were in the form of a reinforcement of the souslik’s colony, implemented in the period 2017-2019. The conservation action was accompanied by a multidisciplinary study of the adaptation process, including investigation of the associated stress, helminth load and spatial behaviour (Kachamakova et al. 2020, Kachamakova and Koshev 2021) and yearly monitoring of the abundance and size of the colony. The monitoring was based on mapping and counting of the ground squirrel’s burrows. The current paper gives an overview of the whole process and presents the results of the monitoring in the period 2017-2021. The aim is to assess the success of the translocation, analyse the factors that influence it and give recommendations for improvement of such actions in future.

## Materials and Methods

### Study area

The location of the reinforced colony is part of the Natura 2000 Sites of Community Importance (SCI) BG0000219 “Derventski vazvishenia” and Special Protection Areas (SPA) BG0002066 “Zapadna Strandzha”, situated in south-eastern Bulgaria. It is near the village of Momina tsarkva (area of 6-7 km^2^, 42.151 N, 27.006 E, 300 m a.s.l.). It consists of pastures with low vegetation height that are regularly grazed by sheep. The area hosts a considerable diversity of predators of *S.citellus*, especially with regards to the raptors - there are data for at least five species of raptors inhabiting the target area - the Eastern Imperial eagle (*Aquilaheliaca*), the Common (*Buteobuteo*) and Long-legged buzzard (*Buteorufinus*), the Booted eagle (*Aquilapennata*) and the Lesser spotted eagle (*Clangapomarina*) (Iankov 2007). In this region of the country, *S.citellus* shares the grassland habitats with one of the largest vole species that occurs in Europe - the Harting's vole (*Microtushartingi*). It is the only social vole and it lives in colonies that are often comprised of over a few dozen individuals and can cover several square metres with densely located burrows (Tabur et al. 2015, Kryštufek et al. 2018).

The donor colony is situated 75 km north of the study area, near Topolchane (42.668 N, 26.437 E, 183 m a.s.l.). It is a vast pasture, grazed by sheep, horses and cattle. It is one of the largest known colonies of the souslik and it has one of the highest genetic diversity (Říčanová et al. 2013). The area is only partly and weakly protected as part of the SPA "Sinite kamani - Grebenets" BG0002058 and some areas were even ploughed during the study period (Koshev et al. 2019).

### Reinforcement process

The initial size of the Momina tsarkva colony was estimated at about a 20-30 individuals with decreasing density and area. The land where the translocated individuals were released has been purchased by the Bulgarian Society for the Protection of Birds (BSPB) to ensure its long-term protection. For the translocation action, a new soft method was applied – the animals were released in individual adaptation cages. Artificial holes were made, feeding and guarding were provided during the first 7-10 days after release. During 3 years, in total 213 ground squirrels were translocated in 2-3 sessions each year during the periods of 21.06 and 19.07 in the years 2017-2019. Their distribution in the sex-age classes is presented in Table 1. Each translocated or captured individual was tagged with an Animal Microchip Syringe encased in 12/2 mm biodegradable glass. Recapture sessions were organised monthly. The stress levels, spatial behaviour and parasite loads were monitored and most of the results are published (Kachamakova et al. 2019, Kachamakova and Koshev 2021) or in preparation. Аt every first animal capture during both translocation efforts and recapture sessions, standard morphometric measurements were taken with a vernier caliper with 1 mm accuracy, except for some cases where the measurement was not possible due to technical issues. The individuals in reproductive state were assigned as adults and those born in the current year - as juveniles. The individuals that did not belong to either of these two groups were assigned as subadults. These were very few and, thus, were not included in the statistical analysis.

### Burrow holes mapping and counting

Every year in the beginning of May, the transect method was applied in order to estimate the number of burrows in the colony. During this time, the colonies of the *Microtushartingi* were also recorded. This method is frequently used for abundance and population density assessment of the *Spermophilus* species (Cepáková and Hulová 2002, Katona et al. 2002, Matějů 2008, Rammou et al. 2021) and is based on the correlation between the number of burrow entrances and animals' abundance (Harper and Batzli 1996, Hubbs et al. 2000). The burrows of the *S.citellus* could be mistaken with those of the European bee-eaters (*Meropsapiaster*) and the hamsters’ (*Cricetuscricetus* and *Mesocricetusnewtoni*), but these species are absent in the study area. It is possible for the Harting's voles’ burrows to be wrongly identified as holes of *S.citellus*, but in general, these are smaller (4-5 cm in diameter compared to ground squirels' holes that are 5–10 cm wide Ramos-Lara et al. 2014), shallower (20-45 cm depth) and densely aggregated (Ondrias 1965). The holes were used to calculate the area of the colony for each year through a “Concave Hull (k-Nearest Neighbour)” tool in QGIS software (QGIS Desktop 3.10.5 with GRASS 7.8.2) buffered with 30 m around the most distant hole.. This value was based on the authors' observations of the mean distance between the consecutive burrows used by a single individual (Kachamakova and Koshev 2021).

### Climate data

The following meteorological data for the period 2017-2021 were obtained from the National Institute of Meteorology and Climatology for the nearest meteorological station - Elhovo, 35 km west of the release site: monthly amount of precipitation in mm (l/m²); number of days of precipitation for the period; number of days with precipitation and monthly amount of precipitation in which the average daily temperature is above 10°С; average monthly air temperature in °С; average monthly maximum and minimum air temperature in °С; monthly absolute maximum and minimum air temperature in °C; number of days and temperature with average daily temperatures above 10°С; number of days with snow cover; maximum monthly height of the snow cover in cm. Selyaninov hydrothermal coefficient (HTC) (Selyaninov 1958) has been used to characterise moisture saturation. It is calculated for the growing season of crops with a biological minimum of 10°C, i.e. for the period April - September, according to the formula: HTC = Sr / 0.1×St, where: HTC - hydrothermal coefficient of Selyaninov; Sr - sum of precipitation for the period with average daily temperatures > 10°С; 0.1 - equivalence coefficient; St - sum of average daily air temperatures > 10°С for the period. The values given by Seleaninov of HTC < 0.5 indicate drought and > 2.0 - overwetting. These values have been adapted according to Bulgarian conditions to an eight-point scale from 0.3 (very dry or arid) to over 1.6 (very humid) (Vlăduţ et al. 2017). The Heinrich-Walter graphical method (Ishida et al. 2013) was used for additional analysis.

### Statistical analysis

The morphometric parameters showed normal distributions and were compared between the translocated individuals and those born in Momina tsarkva, through MANOVA analysis:

- amongst the juveniles captured in the same period of the year, controlling for the sex.
- amongst the adults in both populations, controlling for the sex.

ANOVA analysis was applied to test the difference in the weight amongst the individuals translocated in different years (2017, 2018 and 2019), controlling for the sex and the age. In addition, a Tukey pairwise test was performed to check the differences between the years.

The Pearson’s test was used to check correlation between the number of ground squirrels’ holes and the number of Harting’s voles’ colonies for the period 2018-2021. In 2017, the colonies of *M.hartingi* were scarce and were not counted. In order to assess the relationship in the spatial distributions of the European ground squirrels' holes and Harting's voles' colonies, a 100×100 m grid was created and the number of holes and colonies per square was calculated for each year. Afterwards, a generalised linear model (GLM) was used to test the significance of the interaction between these two variables. Therefore, the number of European ground squirrels' holes in each square were asigned as a dependent variable and the number of Harting's voles' colonies in each square - as an explanatory variable. Due to the overdispersion - the residuals’ deviance was greater than the residuals’ degrees of freedom - a quasi-poisson distribution was applied. These statistical calculations were performed in R-software (R Development Core Team, Version 4.1.0 2021-05-18). In addition, the QGIS software was used for the Nearest-Neighbour analysis to test whether the burrows were randomly distributed or aggregated. The null hypothesis in the Nearest-Neighbour analysis is the random distribution.

## Results

### Morphometry of the translocated and resident individuals

**Figure 1.**
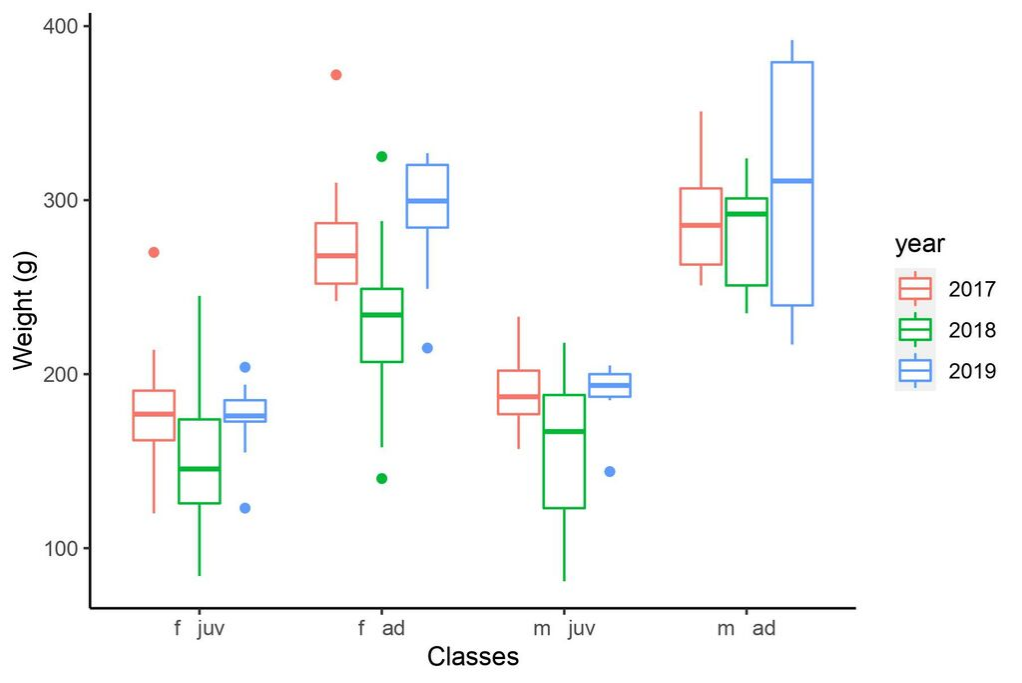
Weight of the different sex-age classes of the individuals translocated to the colony of Momina tsarkva in each year between 2017 and 2019, f - female, m - male, ad - adult, juv - juvenile.

The mean values of the morphometric measurements of each group are presented in Tables 2, 3, 4, and the weights in Fig. 1.

Head (F = 9.09 p = 0.003) and hind-foot length (F = 13.91, p < 0.001) were significantly larger for the juveniles born in the Momina tsarkva colony compared to those from Topolchane according to the results of the multivariate analysis when controling for sex (Table 3). The differences in the weight (F = 0.32, p = 0.571) and the tail length (F = 1.38, p = 0.242) were not significant. The hind-foot length were significantly different between the sexes (F = 8.23, p < 0.005), the difference in head size was close to significant (F = 3.73, p = 0.055,) whereas the weight and the tail length were not (weight - F = 0.14, p = 0.710, tail length - F = 2.47, p = 0.118).

MANOVA analysis showed that the adult individuals from Topolchane were significantly smaller compared to those from Momina tsarkva, based on all three morphometric elements - head (F = 7.89, p = 0.006); tail (F = 5.11, p = 0.026) and feet (F = 8.19, p = 0.005) (Table 2, Table 4). The weight was not tested as the adult individuals were captured in Topolchane only between 18.06 and 19.07 each year, whereas the recapture sessions in Momina tsarkva were held during the whole active season.

The weight varied significantly between the individuals translocated in different years (Fig. 1) when controlling for the sex and the age according to the ANOVA analysis (F = 5.261, p < 0.001). Tukey pairwise comparison showed that the animals translocated in 2017 and 2019 were significantly heavier than those translocated in 2018 (2018-2017: p = 0.003; 2019-2018: p = 0.024). There was no difference in weight between the individuals translocated in 2019 and 2017 (p = 0.922). The other morphometric variables did not show significant differences amongst years.

### Dynamics of the European ground squirrel's burrow holes and colony area. Dynamics in the Harting's vole's colonies

**Figure 2.**
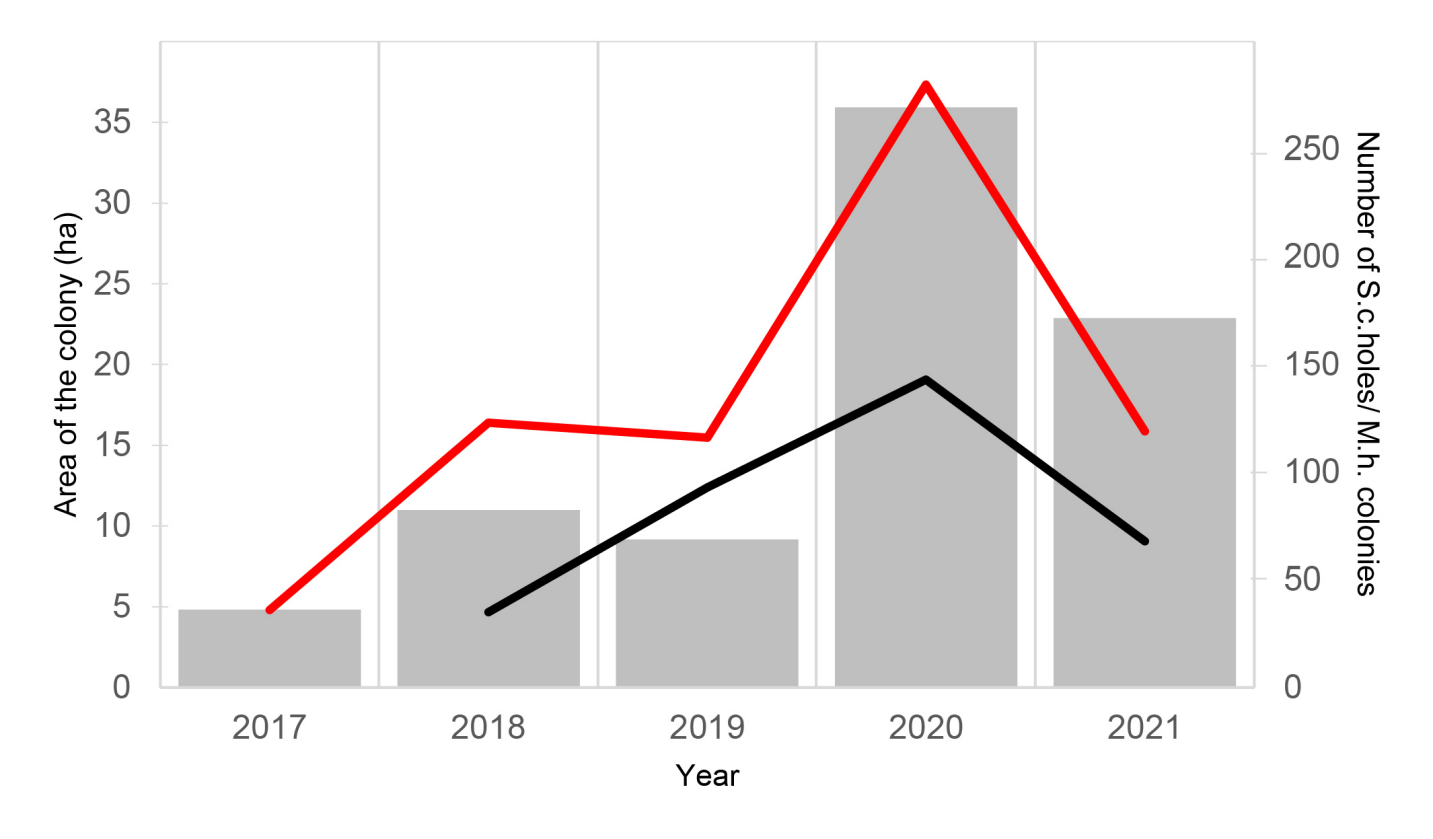
The dynamics in the area of the Momina tsarkva colony (grey bars), the number of European ground squirrel's holes (red line) and the number of Harting's voles' colonies (black line) during the study period (2017-2021).

The burrow number of both species’ colonies increased almost 8 times from 36 in 2017 to 280 in 2020. A decline followed and only 119 holes were recorded in 2021 (Fig. 2). The change in the occupied area of the colony followed the same pattern (Fig. 3). A correlation between the burrow holes of *S.citellus* and the colonies of *M.hartingi* can be seen on Fig. 2, but the Pearson's test was not significant (r = 0.83, p-value = 0.166), probably due to the very small sample size - data for only 5 years are present.

The GLM shows that a statistically significant relationship exists between the spatial distribution of the Harting's vole's colonies and the ground squirrel's burrow holes in 2020 (B = 0.05, t = 5.543, p < 0.000) and 2021 (B = 0.32, t = 2.565, p = 0.015). The analysis did not show significant relationship for 2018 (B = 0, t = -0.006, p = 0.996) and 2019 (B = 0.08, t = 0.517, p = 0.612).

The *S.citellus* holes’ locations were aggregated - the Nearest-Neighbour index was smaller than 1 and the z-score was negative for each year (Table 5).

### Meteorological factors

**Figure 4.**
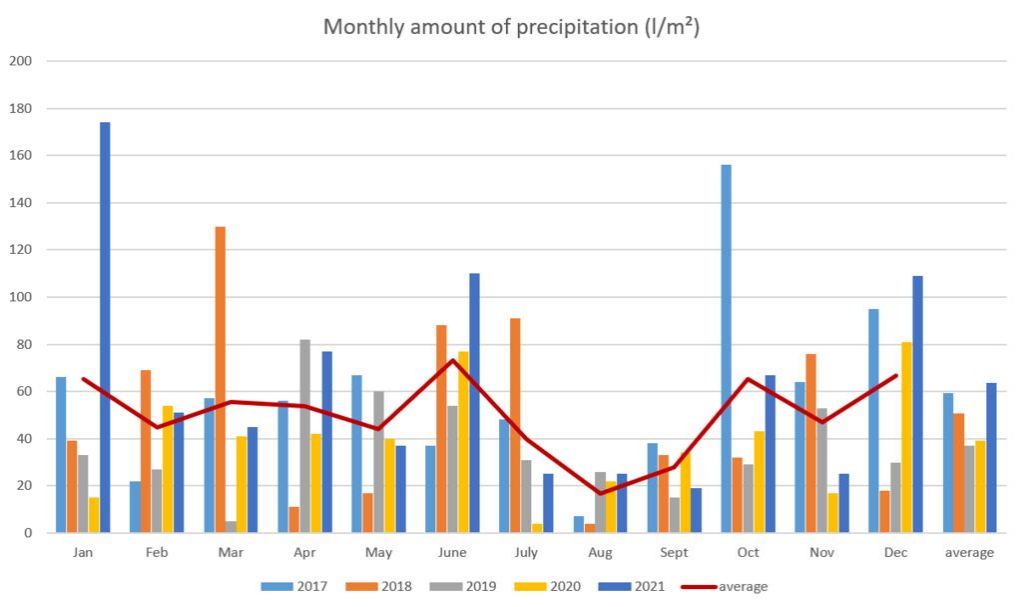
The monthly amounts of the precipitations during the study period (2017-2021).

The monthly amount of precipitation during the study period varied widely due to the unevenly distributed downpours characteristic for the sub-Mediterranean climate (Fig. 4). The precipitation during January 2021, March 2018 and October 2017 highly exceeded the mean monthly value of the other four years (4.6, 3.5 and 3.6 times excess, respectively).

**Figure 5.**
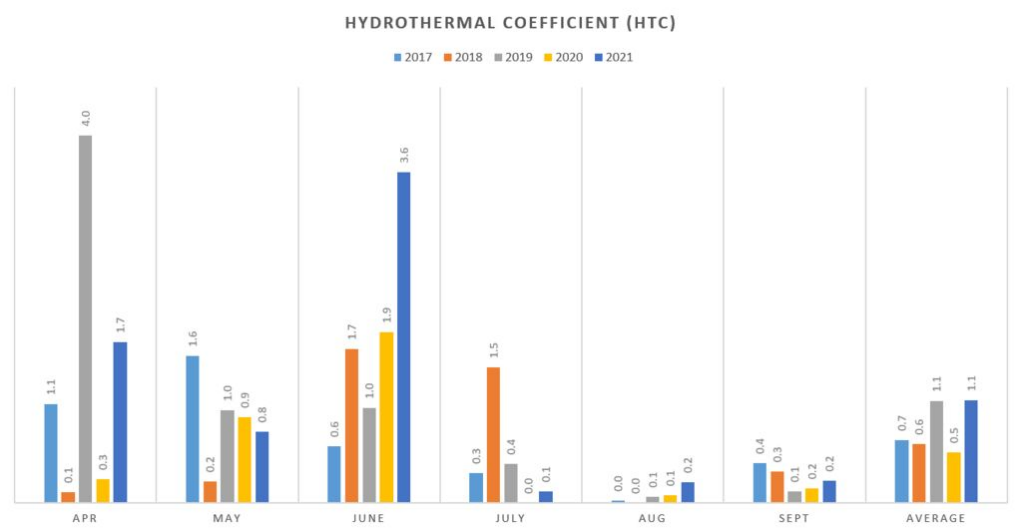
The values of the hydrothermal coefficient in the area of Momina tsarkva for the period (2017-2021).

The hydrothermal coefficient (HTC) showed that the period between July and September was the driest. The fluctuations in HTC were strongest in spring, especially April and June. The years 2017, 2018 and 2020 were dry or moderately dry, while 2019 and 2021 were slightly humid (Fig. 5). The Heinrich-Walter diagrams showed droughts in the period June-September in 2017, 2019, 2020 and 2021, which is normal for this season.

## Discussion

The results suggest that the demographic outcome of the implemented conservation translocation covers most of the criteria to be defined as successful - survival (I), settlement (II) and reproduction (III) of the translocated individuals. A certain level of population growth (IV) is also present, but the population decline in 2021 puts into question the definitive establishment of a viable population (V). Hereafter, we consider all the possible factors that could influence the outcome of the population reinforcement in the Momina tsarkva colony.

### Assessment of the conservation translocation design

Studies have shown that the presence of a resident population, even a small one, increases the chances of survival and success of the conservation translocation and, thus, the reinforcements are more successful than the re-introductions and introductions (Koshev et al. 2019). Тherefore, the translocation in the Momina tsarkva colony has higher chances of a successful outcome. The duration of the translocations also impacts the success rate - 79% of the projects that lasted less than two years lead to negative population growth (Morris et al. 2021). In the current project, the duration of three years was chosen for two main reasons. First, the early released individuals improve the habitat by developing and enlarging the net of burrows that directly increase the chances for survival for the next cohort. Second, it gives the population a better chance to overcome possible short-term stochastic demographic or environmental events that could easily cause fluctuations in the number of individuals (Bodenheimer 1949, Çolak et al. 1998, Atanasova et al. 2010, Krebs 2013). The observed drop in the numbers of ground squirrel's burrow and vole's colonies in 2021 is probably an example of such an event even with translocations occurring in more than two years.

The number of translocated individuals is positively correlated with a successful outcome (Morris et al. 2021). In the current reinforcement, it was chosen well beyond the number reported as optimal (minimum 60 in total and at least 23 per season - Matějů et al. 2012). The aim was to guarantee the success and to save more animals from the donor population in an area that was progressively ploughed during the observed period (Koshev et al. 2019).

During the second and the third year of the action, the number was reduced, as the evidence suggests that the individuals of the supplementary sessions show higher survival rates compared to the pioneers (Swaisgood et al. 2019). Regarding the method of release (soft vs. hard), the decision to apply the soft method (comprising adaptation enclosures, artificial burrows, additional feeding and guarding during the first 7-10 days) was in accordance with the conclusions of the experience gained in the northern part of the species’ range (Matějů et al. 2012, Koshev et al. 2019). Although a recent review of a considerably larger dataset of translocations of amphibians, reptiles, birds and mammals failed to prove the existence of a correlation between the translocation success and the release method (Morris et al. 2021), three previous studies found such a relationship, including data on *S.citellus* (Fischer and Lindenmayer 2000, Gedeon et al. 2011, Matějů et al. 2012).

The analysis of the morphometric measurements shows that both the juvenile and the adult resident individuals are larger than translocated ones. It is unlikely that this difference is genetically based because, according to Říčanová et al. (2013), the two populations share two of three genetic lineages. A possible reason for the observed differences could be the high level of intra-specific competition in the Topolchane colony, where the population density is notably greater. We did not observe any significant differences in weights of the juveniles between the two populations or between the sexes. This suggests that, for the juveniles, the body condition, physiological state, litter size and history are more important than factors like sex and population identity in terms of weight. In addition, the weight is very much related to the progression of the active season, as the individuals translocated in 2018 were significantly lighter that those translocated in 2017 and 2019 when accounting for age and sex. It was most probably due to the fact that, in 2018, the translocation started earlier (21.06-19.06) than in 2017 (06-19.07) and 2019 (01-17.07).

### Behaviour issues

Behaviour issues, especially long-distance dispersal away from the release site, are the most common problem reported during conservation translocations (Berger‐Tal et al. 2020). In order to mitigate and monitor this issue in the framework of the current population reinforcement, a set of measures was applied including radio-collaring part of the released animals (40 out of 213 or 18.78%) during two years (2017 and 2019). As a result, detailed information about the spatial behaviour of the animals was gained (Kachamakova and Koshev 2021). The territories of the translocated individuals were larger than those of the residents and the maximal observed distance passed was considerable - 750 m. However, most of the individuals settled relatively close to the point of release (on average 113 m) and had a very high survival rate (79%) after the first two months of the translocation. This is higher compared to post-translocation data on other ground squirrel species in northern America showing survival of 20%-40% (Swaisgood et al. 2019) and 40-70% (Shier and Swaisgood 2012) after the first 3 months in the new environment.

### Physiological issues - stress

The reinforcement process inevitably leads to an increase in the stress levels of the translocated animals that could hinder their further adaptation and survival (Teixeira et al. 2007, Dickens et al. 2010). Stress could be the root cause for many of the behavioural issues that represent the main difficulty during the implementation of conservation translocations (Berger‐Tal et al. 2020). Taking this into account, we assessed the levels of the faecal cortisol metabolite (FCM) in the resident and translocated individuals – a non-invasive technique proved to give reliable information about the stress experienced by the animals (Möstl and Palme 2002). Although the stress was elevated in the first days after the translocation, it dropped afterwards during the process of adaptation and most probably was not associated with lower survival rates (Kachamakova et al. 2020). In addition, the analysis showed no significant effect of FCM concentration on dispersal distance.

### Possible helminth parasite infection

Disease and parasites present a difficulty in nearly 15% of the conservation translocation efforts, according to a review, based on 293 case studies all around the world (Berger‐Tal et al. 2020). Taking into account this threat we applied a non-invasive method (collection of faecal samples and subsequent analysis through a flotation technique, following Cringoli et al. (2010)) to investigate the presence of helminth parasites in the resident, translocated and newly-produced individuals during the study period. The results suggest that the donor population exhibited a higher diversity and abundance of helminths that were transmitted to individuals from the Momina tsarkva colony. However, after the emergence from hibernation in the spring, the parasite load was considerably reduced in all individuals (Kachamakova et al., unpublished data). This finding is in accordance with other studies (Cahill et al. 1967, Callait and Gauthier 2000) and suggest that, in this particular case, the co-translocation of the helminths did not affect the success of the conservation action.

### Climate factors

Environmental conditions, like harsh weather, can cause difficulties in nearly 15% of the translocations (Berger‐Tal et al. 2020). In general, European ground squirrels seem to prefer areas with low variation in precipitation seasonality (Rammou et al. 2022), whereas high precipitation values can negatively affect the population densities (Zaharia et al. 2016). Other ground squirrel species also show a similar dependence on weather conditions. For example, the population density of *Spermophiluspygmaeus* is strongly influenced by spring, autumn and winter temperatures and the amount of precipitation, which favours the development of vegetation in January, May, June and August (Okulova et al. 2006). In addition, random extreme events (e.g. natural catastrophes) in colony areas can lead to population declines or even local extinctions, particularly when the number of individuals is small. Such an example was recorded in Olšová Vrata (Czech Republic), when a rapid snow melting in the spring of 2004 accompanied by rainfall caused a sharp decline in European ground squirrel numbers (Matějů 2004). Moreover, torrential rain in 2002 caused mass death and a subsequent decline in ground squirrel numbers at the localities Trhovky, Dublovice - Chramosty and Albeř (Havelík 2002). A similar case was observed in the Ponor Mountain (Bulgaria), where hundreds of drowned ground squirrels were found in a cave after heavy rains in the area (Stoyanov 2001). In the present study, a sharp decline in the abundance of both ground squirrels and Harting’s voles in 2021 coincided with the unusually high rainfall in January (174 l/m^2^), which was almost 5 times above the average for the 5-year period. Further investigation of the data showed that these huge amounts of precipitation took place in combination with the highest maximum temperature for January of 17.4°C. Other months with abnormally heavy rainfall were also recorded during the 5-year period. One was in October 2017 after a drought period, when the soil was dry and absorbed the water better. The other case was in March 2018, as the animals begin to be more active and able to leave the burrows, thus avoiding drowning (Fig. 4).

The hydrothermal values during these years do not show any unusual abrupt changes. The lack of moisture is more limiting than the heat for the germination, growth and yield of grass vegetation. Moisture supply is an important factor showing the conditions for the development of the vegetation which the ground squirrels feed on. At low HTC, a decrease in the population numbers has been reported (Koshev 2012). However, in the study area, such a phenomenon was not observed. The lack of excessive HTC values during the study period, despite the considerable variations in the precipitation, could be due to the fact that this coefficient reflects the climate conditions only during the vegetation season (April-September), whereas the excessive precipitation values were outside this period.

### Interspecific interactions and population cycles

The spatial correlation between the burrow systems of *S.citellus* and *M.hartingi* suggest that they do not just share preferences for open habitats (Kryštufek and Vohralík 2005), but also seem to have similar microhabitat requirements. European ground squirrel's burrow aggregations could be influenced by microtopographic characteristics (Katona et al. 2002, Gedeon et al. 2021), such as soil type, thickness and content, slope exposure, presence of trees and bushes. However, no data were found in literature on the interactions between these two social rodents. A recent study reported spatial avoidance between the European souslik and other ground dweling mammal - the European mole, *Talpaeuropaea* (Łopucki et al. 2022). Our observations suggest that it is possible that *S.citellus* occasionally predates on *M.hartingi* – in the Momina tsarkva colony in June 2020, a decapitated body of *M.hartingi* was found in front of a ground squirrel’s burrow. However, this is a single occasion for the entire 5-year study and the act of killing was not observed. The fact that, during the study period, both species simultaneously exhibited an increase in population numbers followed by a drop in 2021 shows that their interactions are not antagonistic, but rather they depend on the same factors. Similarly, co-existence was studied in the meadow steppes of northeast China between *Spermophilusdauricus* and *Microtusgregalis* (Shuai et al. 2014). The study demonstrates that, despite the interspecific competition (resulting in disadvantageous effects to the voles), the activity patterns of *M.gregalis* are correlated with the ambient temperature of the environment, rather than with the presence of *S.dauricus*. *Spermophilusdauricus* was also observed to kill *Microtusgregalis*, but not to feed on it.

Voles are known to exhibit population cycles every 3-5 years (Krebs 2013). Atanasova et al. (2010) found that the average density of *M.hartingi* in the years 2002, 2005 and 2007 in several regions of Bulgaria was 15.7 colonies/per 100 m^2^ (min = 4, max = 39), but in 2007, almost all monitored colonies were inactive. These results indicate that the species exhibit sudden depressions in density, which were also observed in the present study. In the target area, the meаn number of *M.hartingi* colonies per 100 x 100 m square also varied greatly through the years: 2.9 in 2018 (0-9, n = 13); 5.5 in 2019 (0-12, n = 18), 7.3 in 2020 (1-45, n = 39) and 2.1 in 2021 (1-6, n = 33). Contrary to the small rodents, population cycles were not found in *S.citellus* (Danalia 1982). However, variations in the population densities over the years have been observed (Hoffmann et al. 2003, Hoffmann et al. 2003, Ramos-Lara et al. 2014). In one population, the reported abundance of non-juvenile *S.citellus* ranged between 110 individuals per ha in 1993 and 6.5 per ha in 1998 (Hoffmann et al. 2003). There was a decrease in density by 5.9 times, until the complete disappearance of the colony (Hoffmann et al. 2003, Hoffmann et al. 2003).

### Conclusions and proposal for nature conservation

Despite being considered for decades (Sutherland et al. 2004) and widely approved, the evidence-based approach in conservation is still not applied in many cases. The aim of the current study is to give a detailed overview of a conservation action and the follow-up monitoring that can be directly used by scientists and practitioners in planning and implementing similar measures. We emphasise the need for standardised and long-term monitoring of the populations after the translocation, which is a fundamental part of the translocation process (IUCN 2013). However, in one of every three cases, wildlife managers had difficulty with the post-release monitoring of released individuals (Berger‐Tal et al. 2020). This hinders the accumulation of knowledge and the progress of positive outcomes obtained through experience and improvement (Morris et al. 2021). Our results indicate the initial success could be temporary and considerable fluctuations could emerge in the population’s size and range. These could be due to stochastic events, for which understanding further monitoring and analysis are needed. Yet at this point, our results show that the survival of the translocated individuals is high, the reproduction is present and, despite the drop in 2021, the population numbers in the Momina tsarkva colony remain higher than the initial population size. Thus, the conservation measures could be considered successful. The monitoring will be continued during the following years and will demonstrate if the observed drop will be reversed or will deepen.

## Figures and Tables

**Figure 3a.**
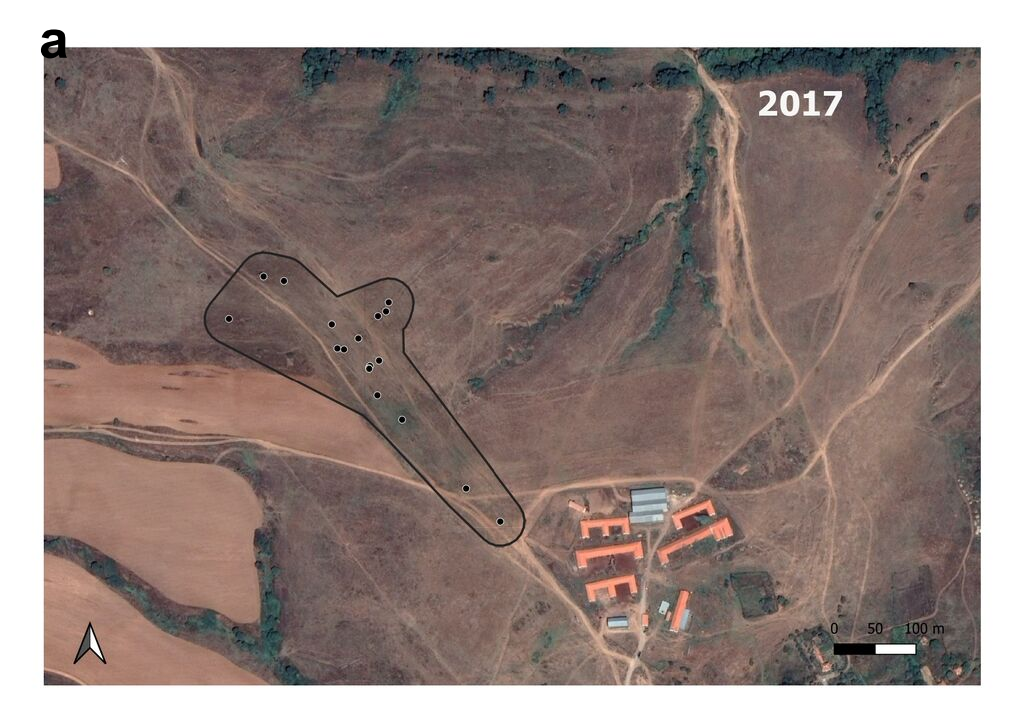
In 2017; the *M.hartingi*’s colonies were scarce and were not mapped.

**Figure 3b.**
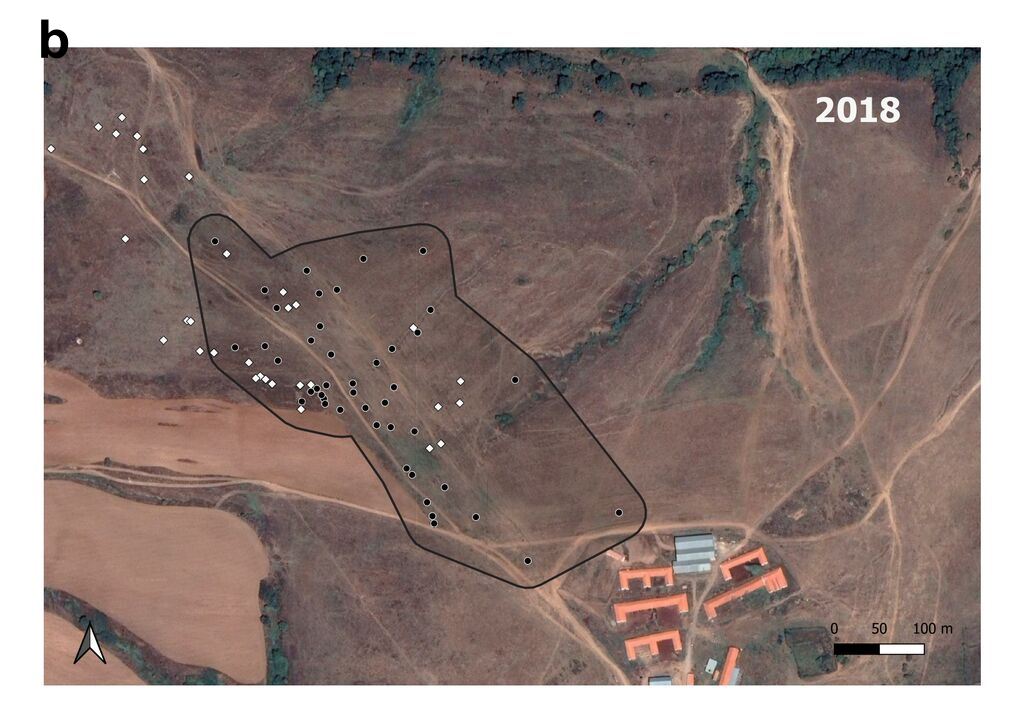
In 2018

**Figure 3c.**
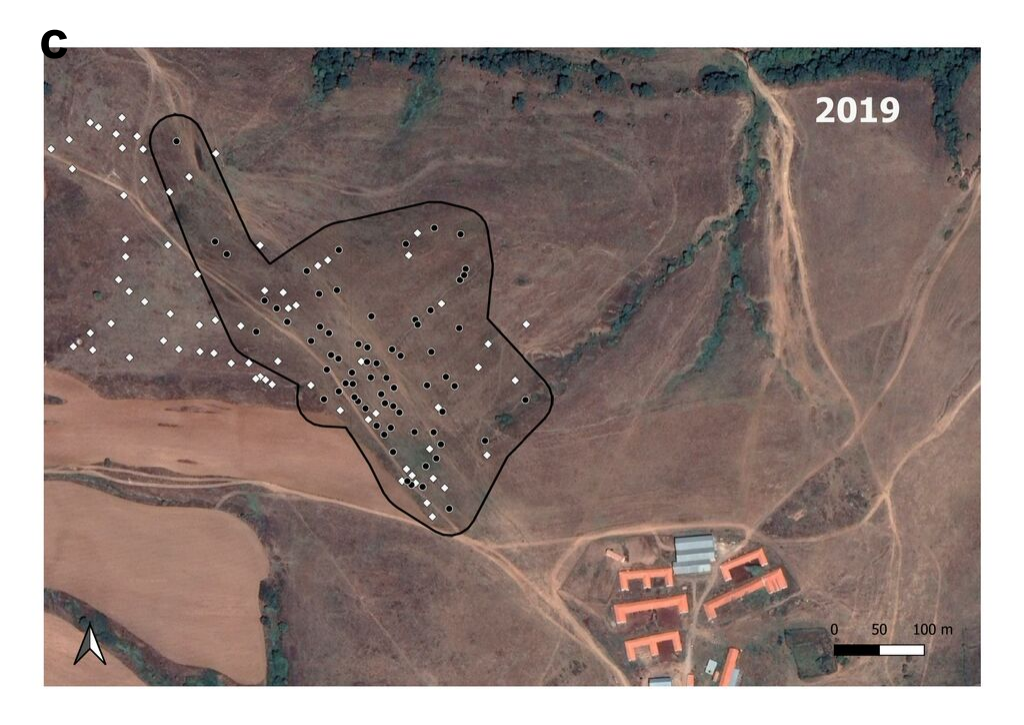
In 2019

**Figure 3d.**
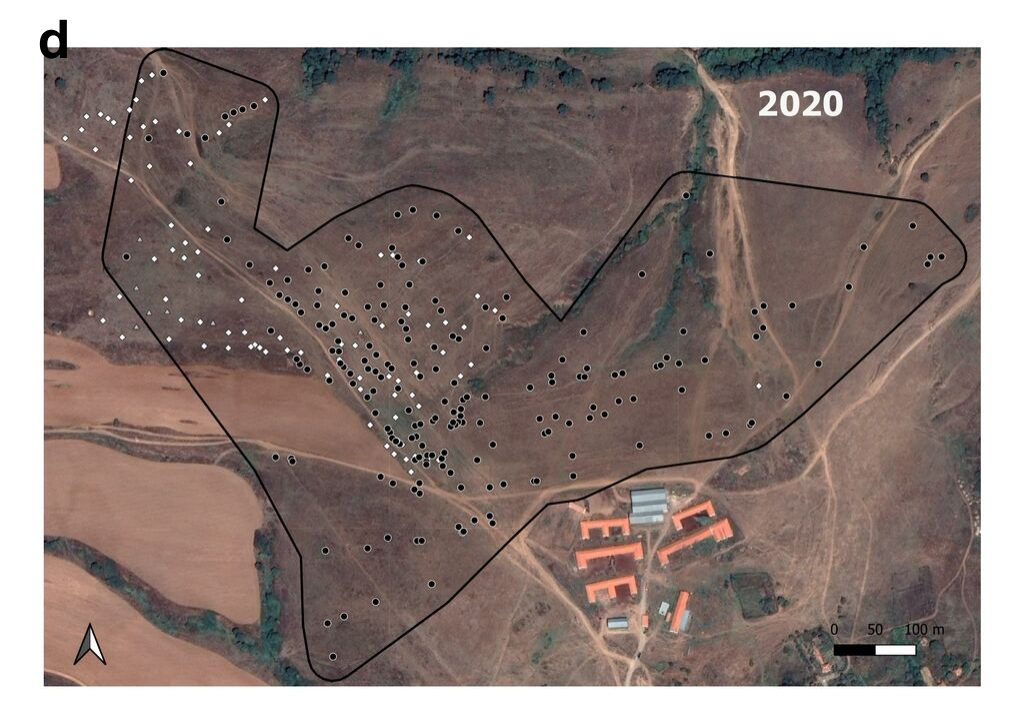
In 2020

**Figure 3e.**
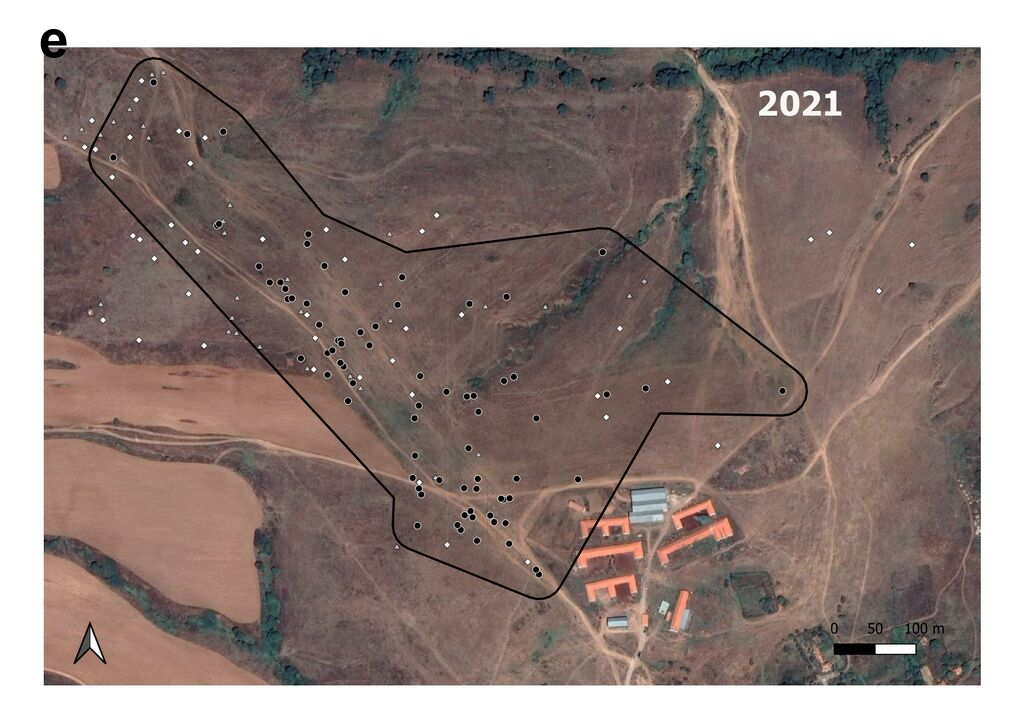
In 2021

**Table 1.** Sex and age of the translocated individuals throughout the three years of the population reinforcement action. Non-reproducing includes juveniles and subadults.

Release sessions	Non-reproducing		Reproducing		Total
	Males	Females	Males	Females	
6 July 2017	2	18	9	3	32
13 July 2017	5	13	4	6	28
19 July 2017	14	12	8	2	36
21 June 2018	8	8	2	9	27
4 July 2018	4	5	5	3	17
19 July 2018	11	5	6	5	27
1 July 2019	4	6	2	1	13
17 July 2019	8	14	2	9	33
**Total**	**56**	**81**	**38**	**38**	**213**

**Table 2.** Mean values of the standard morphometric measurement of each sex-age group of the translocated adult ground squirrels in the period 2017-2019. n = sample size, SD = standard deviation.

	Weight (g)	Head size (mm)	Tail length (mm)	Hind-foot length (mm)
ad m	288 (SD = 41, n = 29)	52 (SD = 3, n = 38)	58 (SD = 8, n = 38)	35 (SD = 2, n = 38)
ad f	260 (SD = 50, n = 35)	50 (SD = 3, n = 38)	55 (SD = 7, n = 37)	34 (SD = 2, n = 38)

**Table 3.** Mean values of the standard morphometric measurements of the resident juveniles captured in Momina tsarkva (R) between 21.06 and 19.07 of each year (2017-2019) and the juveniles from Topolchane (T), translocated in the same period. n = sample size, SD = standard deviation

	Weight (g)	Head size (mm)	Tail length (mm)	Hind-foot length (mm)
T juv m	173 (SD = 37, n = 36)	49 (SD = 3, n = 38)	59 (SD = 6, n = 38)	34 (SD = 1, n = 37)
T juv f	168 (SD = 34, n = 51)	47 (SD = 3, n = 64)	56 (SD = 7, n = 62)	33 (SD = 2, n = 64)
R juv m	166 (SD = 42, n = 26)	49 (SD = 3, n = 26)	60 (SD = 7, n = 26)	35 (SD = 2, n = 26)
R juv f	167 (SD = 32, n = 26)	49 (SD = 2, n = 30)	59 (SD = 5 n = 30)	35 (SD = 2, n = 30)

**Table 4.** Mean values of the standard morphometric measurements of the adult ground squirrels captured in Momina tsarkva throughout the year (R) and of those translocated from Topolchane (T). n = sample size, SD = standard deviation

	Head size (mm)	Tail length (mm)	Feet size (mm)
T ad m	52 (SD = 3, n = 38)	58 (SD = 8, n = 38)	35 (SD = 2, n = 38)
T ad f	49 (SD = 8, n = 38)	55 (SD = 7, n = 37)	34 (SD = 2, n = 38)
R ad m	53 (SD = 3, n = 9)	60 (SD = 11, n = 9)	36 (SD = 1, n = 9)
R ad f	52 (SD = 2, n = 21)	60 (SD = 5, n = 21)	35 (SD = 2, n = 21)

**Table 5.** Results of the aggregation analysis of the European ground squirrel's holes mapped in the period 2017-2021 show significant aggregation for each year - the Nearest-Neighbour index is smaller than 1 and the z-score values are negative.

Year	2017	2018	2019	2020	2021
z-score	-2.799	-0.895	-3.080	-10.412	-6.085
Nearest-Neighbour index	0.655	0.929	0.806	0.617	0.637
